# Acknowledgement to Reviewers of the *Journal of Clinical Medicine* in 2013

**DOI:** 10.3390/jcm3010216

**Published:** 2014-02-24

**Authors:** Journal of Clinical Medicine Editorial Office

**Affiliations:** MDPI AG, Klybeckstrasse 64, CH-4057 Basel, Switzerland

The editors of the *Journal of Clinical Medicine* would like to express their sincere gratitude to the following reviewers for assessing manuscripts in 2013:
Almog, NavaDrago, DeniseHirai, YujiAmadori, DinoEconomou, AthinaHoussiau, Frederic A.Antebi, BenEsteban, AndrésHubalek, MichaelBernal-Mizrachi, CarlosEvans, MarkIngvild, IngvildBourin, P.Facchini, AndreaJosson, SajniBouza, EmilioFong, Daniel Tik-PuiKasten, Mary J.Bove, GeoffreyForoozan, RodKim, Han SuBower, ChrisFrölich, J.Kohn, Michael R.Brodowicz, ThomasFüllen, GeorgKusuma, SravantiBroggini, MassimoGalanter, Cathryn A.Kwon, Tae GyunBrufsky, AdamGoetzl, LauraLanguino, LuciaCalifaretti, NadiaGoldstein, Benjamin I.Laurie, Karen L.Casimiro, SandraGräs, SørenLee, Moon SooCavoretto, PaoloGunetti, MonicaLi, GangConnelly, KimHagmann, SébastienLipton, AllanConover, JoanneHalperin, JohnLu, BaisongCousens, NicoleHarper, GordonLungu, CodrinCunha, Burke A.Hashmi, FarinaLuparello, ClaudioDahlström, Lisen ArnheimHaubitz, SebastianLutz, StephenDavies, Robert D.Hematti, PeimanMagann, Everett F.De Geest, BartHenry, DavidMccloskey, EugeneDennis, KristopherHernigou, PhilippeMlineritsch, BrigitteMontgomery, ScottRincón, Olga Gómez delTakebe, NaokoMorris, Patrick G.Rodriguez, Anne-marieTeich, StevenMortara, FrancoRosenblatt, Jon E.Trial, JoannNarod, StevenSalgado, A. J.Truong, DanielNgim, Chin FangSchul, WouterVagefi, RezaNilar, ShahulSchuleri, KarlVan Poznak, CatherineOBrien, LouiseSelvaraj, VimalVerhagen, EalmOdejinmi, F.Sesti, FrancescoWang, QunPadfield, Gareth J.Shaak, Thomas L.Wang, YunPark, WonSonne, Susan C.Watanabe, ShigeruPergament, EugeneSprick, Martin R.Wilson, KumananPergolizzi, StefanoSpringett, KateYasuhara, TakaoPolo, JoseSridharan, ShankarYennamalli, RagothamanPontikoglou, CharalamposStoddard, JoelYu, SeongjinPorpora, M. G.Stoeger, HerbertZhang, JianPowles, TrevorSubbulakshmi, SubbulakshmiZhang, YongRadisky, DerekSundar, Krishna M.Zhu, XuegongReed, ElizabethSykes, Jane



